# The Correlation of Serum Growth Differentiation Factor-15 Level in Patients with Obstructive Sleep Apnea

**DOI:** 10.1155/2015/807683

**Published:** 2015-05-04

**Authors:** Kamran Sari, Huseyin Ede, Zeliha Kapusuz Gencer, Mahmut Ozkiris, Ayse Yesim Gocmen, Yavuz Selim Intepe

**Affiliations:** ^1^Otorhinolaryngology Department, Bozok University School of Medicine, 66200 Yozgat, Turkey; ^2^Cardiology Department, Bozok University School of Medicine, 66200 Yozgat, Turkey; ^3^Biochemistry Department, Bozok University School of Medicine, 66200 Yozgat, Turkey; ^4^Chest Disease Department, Bozok University School of Medicine, 66200 Yozgat, Turkey

## Abstract

*Purpose*. Growth differentiation factor-(GDF-) is a prognostic biomarker in cardiovascular disorders (CVD). GDF-15 level was not studied in patients with obstructive sleep apnea syndrome (OSAS) before. In this study, we investigated serum GDF-15 levels in OSAS patients and compared them with healthy controls. *Material and Methods*. Polysomnographically, confirmed forty consecutive OSAS patients (20 men and 20 women) and forty consecutive healthy controls (23 men and 17 women) were enrolled in the study. The samples in each group had similar demographic characteristics and body mass index (BMI) values. *Results*. In the study, no significant correlation was found about GDF-15 levels of OSAS group and healthy controls. However, there was a significant statistical correlation between age and GDF-15 level. In correlation analysis, there was not any significant correlation between age and BMI. *Conclusion*. Although various developing biomarkers have been studied in cardiovascular disorders, GDF-15 levels have attracted a widespread interest as predictors of cardiovascular risk. GDF-15 level has not been evaluated previously in patients with OSAS. A significant statistical correlation was found between age and GDF-15 level. To reveal close relation between OSAS and GDF-15, further studies are needed with combination of GDF-15 and other biomarkers in OSAS.

## 1. Introduction

Obstructive sleep apnea syndrome (OSAS) is characterized by frequent sessions of collapse of upper airways during sleep, resulting in recurrent episodes of intermittent hypoxemia and arousal from sleep [1–3]. Its incidence rises increasingly in the society. It influences approximately 24% of men and 9% of women [4]. OSAS leads to an increase in metabolic and cardiovascular problems if untreated [5, 6]. In a meta-analysis, Wang et al. showed that severe OSAS is significantly and independently associated with an increased risk of cardiovascular disease (CVD), stroke, and all-cause mortality [7]. Takama and Kurabayashi [8] reported that the presence of OSAS was a strong predictor of fatal cardiovascular events in patients with CVD. Growth-differentiation factor-15 (GDF-15) is a secreted protein, which is a member of the transforming growth factor-*β* (TGF-*β*) cytokine superfamily [9]. GDF-15 is not expressed in the adult myocardium at normal circumstances. But its expression is strongly induced in the heart during experimental ischaemia and reperfusion injury [10]. GDF-15 is highly expressed in the placenta and the prostate, bur its level is not normal in many other organs like heart [8, 9]. GDF-15 has recently been defined as a protein that is upregulated in cardiac myocytes by simulated ischaemia, reactive oxygen species, and proinflammatory cytokines [10, 11]. Kempf et al. [12] have recently found that patients with chronic heart failure (CHF) have increased circulating levels of GDF-15. It is also upregulated by some cardiovascular events triggering oxidative stress, heart failure, and atherosclerosis [11, 13].

We hypothesized that serum GDF-15 level may be increased in patients with OSAS because of the relation between OSAS and CVD. In this study, we searched for serum GDF-15 level in patients with OSAS and compared it to that of the patients without OSAS. To the our best knowledge, this is the first study investigating the association between OSAS and GDF-15 level.

## 2. Material and Methods

This prospective, case-control study was performed with cooperation of Otolaryngology, Cardiology, Chest and Biochemistry Departments of Bozok University School of Medicine. Between February 2014 and September 2014, a total of 150 patients were evaluated in suspect of OSAS. Of 150, forty patients who were diagnosed with OSAS were enrolled in the study. The control group consisted of forty healthy samples. All patients and controls were examined by the Ear, Nose, Throat and Chest departments to validate inclusion criteria before the study. Patients were not included in the study if they have had any of the following disease or disorders: presence of CVD, congenital abnormalities of the head and neck region, recent head trauma, neurologic or metabolic diseases (epilepsy, diabetes mellitus), history of previous use of sedatives and muscle relaxants in the last 3 months, sleep disorders other than OSAS (e.g., restless legs syndrome, insomnia), and any pulmonary disorders.

The participants were given information about the study according to Helsinki Declaration. Approval of the institutional ethics committee was obtained from Bozok University School of Medicine Ethics Committee on Non-Interventional Clinical Investigation.

Polysomnographically, confirmed 40 consecutive OSAS patients (20 men and 20 women) with an average age of 48 years (18–63 years) and 40 consecutive control subjects (23 men and 17 women) with an average age of 46 years (29–65 years) were recruited for the study. The subjects in each group had similar demographic characteristics and body mass index (BMI) values. Weight and height measurements were used to calculate BMI (kg/m^2^) values. BMI > 30 kg/m^2^ was defined as obesity. Routine physical and otolaryngological examinations were performed in both groups. Polysomnographic evaluation was found normal in the control group (apnea/hypopnea index (AHI) < 5).

According to the polysomnographic evaluation, an apnea/hypopnea index (AHI) equal or more than five per hour of sleep was used to diagnose OSAS. Apnea was defined if airflow dropped below 10% of the reference amplitude for 10 seconds. If airflow dropped below 70% for 10 seconds with a following oxygen desaturation of 4% or more, it was defined as hypopnea. The AHI index was calculated with the average number of apneas and hypopneas per hour of sleep. At polysomnographic analysis, electrocardiography, oronasal airflow measurement, duration of snoring periods, thoracic and abdominal respiratory movements, body position changes, and oxygen saturation (pulse oximeter) were measured and recorded simultaneously with the sleep stages by electroencephalography, electrooculography, and chin and tibial electromyograms. Sleep stages were scored according to the standard criteria of American Academy of Sleep Medicine (AASM) criteria. Patients with AHI < 5 were evaluated as normal while patients with AHI ≥ 5 were defined as OSAS. An AHI score between 5 and 15 indicated mild OSAS, 16 to 30 indicated moderate OSAS, and ≥30 indicated severe OSAS [14].

## 3. Blood Sampling and Laboratory Analyses

After an overnight fasting, venous blood samples were collected. Within an hour of venipuncture, whole blood samples were centrifuged and separated, and serum portions were frozen at −80°C for future analysis. Serum GDF-15 levels were determined using a commercially available enzyme-linked immunosorbent assay (ELISA) kit (BioVendor, Brno, Czech Republic).

### 3.1. Statistical Analyses

Statistical analyses were performed using the SPSS package programme. Continuous variables are expressed in mean ± SD and categorical variables are presented as frequencies (%). Normality of variables was tested with Kolmogorov-Smirnov test. Age and BMI values did show normal distribution while GDF-15 did not show normal distribution. Thus, Pearson and Spearman tests were used for correlation analyses accordingly, and Mann-Whitney *U*-test and student's *t*-test were used to compare groups accordingly. Categorical variables of the groups were compared using the chi-square test. A *p* value of less than 0.05 was considered to show statistically significant result.

## 4. Results

**Figure 1:**
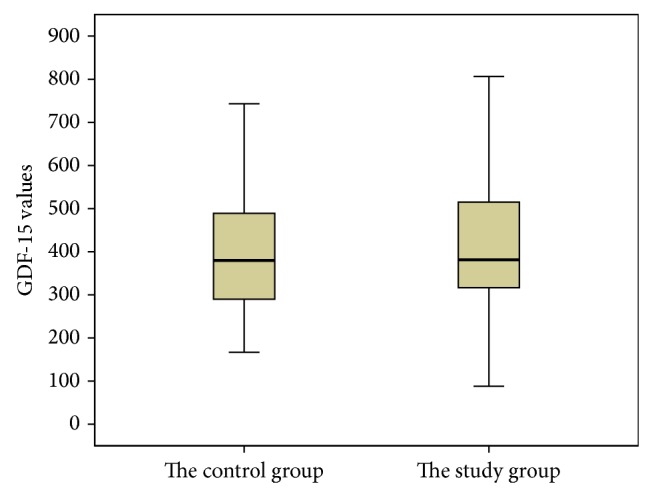
The average GDF-15 levels of OSAS group and control group.

In the study, we did not find any significant difference between OSAS patients and controls in respect to BMI (*p* > 0.05). The control and OSAS group demonstrated average AHI levels of 2,32 and 36,77, respectively. Sixteen patients had severe OSAS (AHI III), twelve patients with moderate OSAS (AHI II), and twelve patients with mild OSAS (AHI I). In correlation analysis, we did not find any significant correlation between age and BMI (*p* = 0.531;  *r* = 0.071). However, there was a significant statistical correlation between age and GDF-15 level (*p* = 0.468, *r* = 0.000). Oppositely, GDF-15 did not show any statistical correlation to BMI (*p* = 0.697, *r* = 0.044). The average GDF-15 levels of OSAS group and control group were found to be 413 ng/L and 410 ng/L, respectively (Figure 1). The demographic characteristics and average level of GDF-15 of the groups were statistically similar and expressed in Table 1.

## 5. Discussion

GDF-15 is a protein that belongs to TGF-*β* superfamily. GDF-15 level is induced by IL-1, TNF-*α*, and TGF-*β* in macrophages in turn. It restricts macrophage activation and inflammation. In addition, injury, oxidative stress and atherosclerosis can increase secretion of GDF-15. Increasing levels of GDF-15 stimulate anti-inflammatory effect on macrophage, antiapoptotic effect on some cancer cells, and antigrowth and antihypertrophic effect at inhibition of remodeling [15]. Although numerous emerging biomarkers have been studied in cardiovascular events, GDF-15 levels have attracted a widespread interest as predictors of cardiovascular risk. It is pointed that GDF-15 level is associated with the risk of death and myocardial infarction independent of clinical variables and other biomarkers [16]. Dominguez-Rodriguez et al. [17] measured the level of GDF-15 and high-sensitivity C-reactive protein (hsCRP) in patients with acute coronary syndrome at admission and again 36 months after admission. They found that the change of hsCRP levels, measured after 36 months, does not estimate major cardiovascular adverse events (MACE) in patients with acute coronary syndrome. But the level of GDF-15 measured, after 36 months, was a significant predictor of MACE in acute coronary syndrome patients. In a study by Bonaterra and colleagues [18] about GDF-15 knockout mice, it was shown that GDF-15 deficiency protects mice against atherosclerosis. They explained that GDF-15 deficiency results in inhibition of atherosclerosis progression in hypercholesterolemic mice. They also stated that IL-6 plays a major role in the progression of atherosclerosis. In the same study, they showed that GDF-15 is upregulated in the atherosclerotic vessel wall. A recent study found evidence that GDF-15 is associated with infarct size in experimental heart attack models [19].

We think that GDF-15 might be a novel strong biomarker in cardiovascular diseases like heart attack or atherosclerosis. And this hypothesis is supported by a recent study on the association of GDF-15 and coronary diseases [20].

OSAS is a common disorder 22% in the general population aged 50–80 years and defined as AHI > 5/h [21]. Its prevalence increases with age. It is known that OSAS can cause cardiovascular and metabolic dysfunctions by inflammatory cytokines upregulation [4]. The period of intermittent hypoxia and carbon dioxide retention creates imbalance in the relation between sympathetic and parasympathetic nervous regulation [22]. This imbalance increases the heart rate and systemic blood pressure (BP) with sympathetically mediated peripheral vasoconstriction [23]. Intermittent chronic hypoxia and oxidative stress can play role in the induction of reactive oxygen radicals production, inflammatory cytokines release, and endothelial dysfunction in patients with OSAS [4]. On long term, OSAS leads to repetitive nocturnal hypoxia and sleep disturbance. This condition induces an increase in cytokine and inflammatory markers [22]. It is accepted that proinflammatory cytokines and oxidative stress increase GDF-15 secretion. Therefore, we thought that chronic hypoxia and oxidative stress caused by OSAS may lead to an increase in the level of GDF-15.

To the best of our knowledge, this is the first study investigating the association between OSAS and GDF-15 level in a case-control study of women and men. In the present study, we investigated the level of GDF-15 in patients with OSAS and healthy controls. We found similar results in each group. Also our data showed that there was a positive correlation between age and GDF-15 level. GDF-15 level has been found to be increased with aging. It can be due to increased incidence of atherosclerosis, heart failure, and other pathologies with aging that trigger oxidative stress. All of these pathologies increase the level of GDF-15. In a study related to GDF-15 level in ST-segment elevation myocardial infarction (STEMI), Kempf et al. [24] showed that GDF-15 levels were independently related to age, male gender, diabetes, smoking, reduced systolic blood pressure, elevated heart rate, and a cardiac troponin T (cTnT) level at presentation. They discovered that GDF-15 is a new biomarker in STEMI that ensures prognostic information about the disease. They found that increasing levels of GDF-15 were associated with increased age. The upper limit of normal level of GDF-15 is less than 1200 ng/L. In our study, GDF-15 levels were found less than 1000 ng/L in both groups. In an another study by Kempf et al. [12], they found that GDF-15 levels also increases in patient with low body mass index and in patients with myocardial infarction, ischemic etiology, higher New York Heart Association (NYHA) functional class, and a reduced left ventricular ejection fraction (LVEF). In our study, we did not find any correlation between GDF-15 level and BMI.

BMI is another factor that affects GDF-15 level. In a study concerning obesity and GDF-15 level, it was found that GDF-15 levels were higher in obese group than in nonobese group [25]. In our study, BMI levels were similar in both OSAS and the control groups (32 kg/m^2^ and 29.9 kg/m^2^, resp.). Average GDF-15 levels were similar between groups and under 1200 ng/L. In recent studies, it was pointed that GDF-15 level is higher in men than women [24]. In our study, GDF-15 level is higher in men than women, but the difference was not statistically significant. The relation between OSAS categories and GDF-15 levels was analyzed, and significant difference was not found in respect to OSAS categories.

In many numerous studies, different kinds of cardiac markers were investigated in OSAS patients [26, 27]. Randby et al. [27] examined High-Sensitivity Cardiac Troponin T (hs-cTnT) level in patients with OSAS. They categorized the subjects according to AHI levels that correspond to no, mild to moderate, and severe OSAS. They found that the proportion of subjects with detectable hs-cTnT levels increased with increasing severity of OSAS. Even so, there was no independent correlation between AHI and detectable hs-cTnT after the correction for all significant univariate covariates. They interpreted that a clustering of cardiovascular risk factors clarifies the observed association between hs-cTnT levels and the severity of OSAS. C-reactive protein (CRP) is a common inflammatory biomarker. It has been synthesized by hepatocytes in response to inflammatory process. Also, CRP could have been synthesized in coronary artery smooth muscle and adipose tissue [28]. In healthy persons, CRP is a predictor in CVD. It has been studied in patients with OSAS. But the results are controversial [29]. According to a recent meta-analysis, it has been shown that CRP was higher in patients with OSA than control group [30]. Moreover, in another meta-analysis, evidence was found that treatment with continuous positive airway pressure (CPAP) could partly suppress inflammation [31]. Hall and colleagues [32] measured level of CRP and myeloid-related protein-8/14 (MRP-8/14) in patients with OSAS and investigated the relation between obesity and inflammatory parameter. They did not find any definitive indication of independent immunological activity between apneas and hypopneas and inflammatory factors. But they found that average oxygen saturation for MRP-8/14 and oxygen desaturation index for CRP stranded statistically significant predictors. Also they found that CRP was strongly correlated with BMI. Interleukin-6 (IL-6) interleukin-1 beta (IL-1b), and tumor necrosis factor-alpha (TNF-*α*) are one of the important atherogenic markers in ischemic stroke patients. OSAS is seen common especially in acute stroke patients. Medeiros et al. [33] found that IL-6 shows a mean rise in stroke patients with OSAS. It was thought that this inflammatory marker may play a role in the mechanism of atherogenic and endothelial dysfunction in the relationship between OSA and cerebrovascular disease. In their study, serum IL-6 levels were independently negative correlated with desaturation index and low SpO_2_ values. There was no relation between inflammatory biomarkers and AHI in their study.

Inami et al. [22] examined the relation between sleep disordered breathing (SDB) and the severity of coronary artery disease (CAD). They analyzed the relation between SDB, coronary atherosclerotic burden, and cardiac biomarkers in stable CAD patients. They showed that N-terminal pro-B-type natriuretic peptide (NT-proBNP) and hs-TnT levels increased in patients with CAD and SDB. In our study, the patients with OSAS did not have CAD. Thus, GDF-15 level may have been fixed within normal range.

In our study, there was not any significant difference between groups in respect to GDF-15 level. One reason for this, the ratio of patients with severe OSAS were 40% in the OSAS group. There would be significant difference between groups in respect to GDF-15 level if all of the OSAS group consisted of patients with severe OSAS. Considering relation of BMI and GDF-15, no significant correlation was found among the subjects, because we matched BMI values of groups in order to remove any effects of obesity on OSAS.

GDF-15 is an important biomarker in cardiovascular pathologies. GDF-15 level has not been evaluated previously in patient with OSAS. In this study, we found similar GDF-15 levels between OSAS group and the control group. However, we found a significant statistical correlation between age and GDF-15 level. To show the relation between OSAS and GDF-15, we suggest further studies with combination of GDF-15 and other biomarkers in OSAS.

## Figures and Tables

**Table 1:** Demographic characteristics and median level of GDF-15 in OSA and control groups.

	The control group (*n* = 40)	The study group (*n* = 40)
Mean age (in year)	46 ± 9	48 ± 10
Gender (male/female) (%)	23/17 (57/43)	20/20 (50/50)
Body mass index (kg/m^2^)	29.9 ± 3.9	32.0 ± 6.2
GDF-15	410 ng/L ± 159	413 ng/L ± 170
